# Antisenescence therapies for age‐related bone loss: Target factors, medicines, biomedical materials

**DOI:** 10.1002/ctm2.70350

**Published:** 2025-06-09

**Authors:** Qiyue Zhu, Menglong Hu, Likun Wu, Erfan Wei, Xingtong Pan, Hao Liu, Yunsong Liu

**Affiliations:** ^1^ Department of Prosthodontics Peking University School and Hospital of Stomatology & National Center for Stomatology & National Clinical Research Center for Oral Diseases & National Engineering Research Center of Oral Biomaterials and Digital Medical Devices& Beijing Key Laboratory of Digital Stomatology & NHC Key Laboratory of Digital Stomatology & NMPA Key Laboratory for Dental Materials Central Laboratory Peking University School and Hospital of Stomatology Haidian District Beijing PR China; ^2^ Central Laboratory Peking University School and Hospital of Stomatology & National Center for Stomatology & National Clinical Research Center for Oral Diseases & National Engineering Research Center of Oral Biomaterials and Digital Medical Devices& Beijing Key Laboratory of Digital Stomatology & NHC Key Laboratory of Digital Stomatology & NMPA Key Laboratory for Dental Materials Peking University School and Hospital of Stomatology Haidian District Beijing PR China

**Keywords:** biomedical materials, bone aging, cellular senescence, medicines, target factors

## Abstract

Progress in living conditions and medical technology have extended the human life span such that population aging, and thus the development of multi‐system degenerative diseases, has become a major problem in many countries. Bone is a metabolically dynamic tissue and bone aging is closely related to a shift in the balance between bone resorption and bone formation. The resulting loss of bone mass and bone mechanical properties in older adults place them at risk of injury and premature death. Cellular senescence occurs in response to endogenous and exogenous stresses that lead to permanent cell cycle arrest and, thus, to tissue degeneration and dysfunction. Senescence in the bone microenvironment, as occurs during aging, induces a decline in bone formation. Research into the treatment of bone aging has therefore focused on the senescence process. This review begins with a summary of the key events in cellular senescence and bone aging and then examines recent progress in the targeting of cellular senescence, both to treat aging‐related bone diseases. Novel therapeutic agents, natural products, and innovative biomedical materials are considered. Our discussion concludes by considering areas of future research.

## Graphical abstract

With extended lifespans causing population aging, bone aging has become a worldwide issue. Cellular senescence in the bone microenvironment causes bone degeneration and dysfunction. This review summarises targets in cellular senescence and bone aging while detailing potential therapeutic strategies, including novel medicines and innovative biomedical materials. https://BioRender.com/0wqyt7g

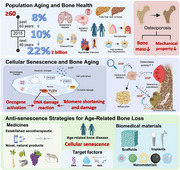

## 1 INTRODUCTION

**FIGURE 1:**
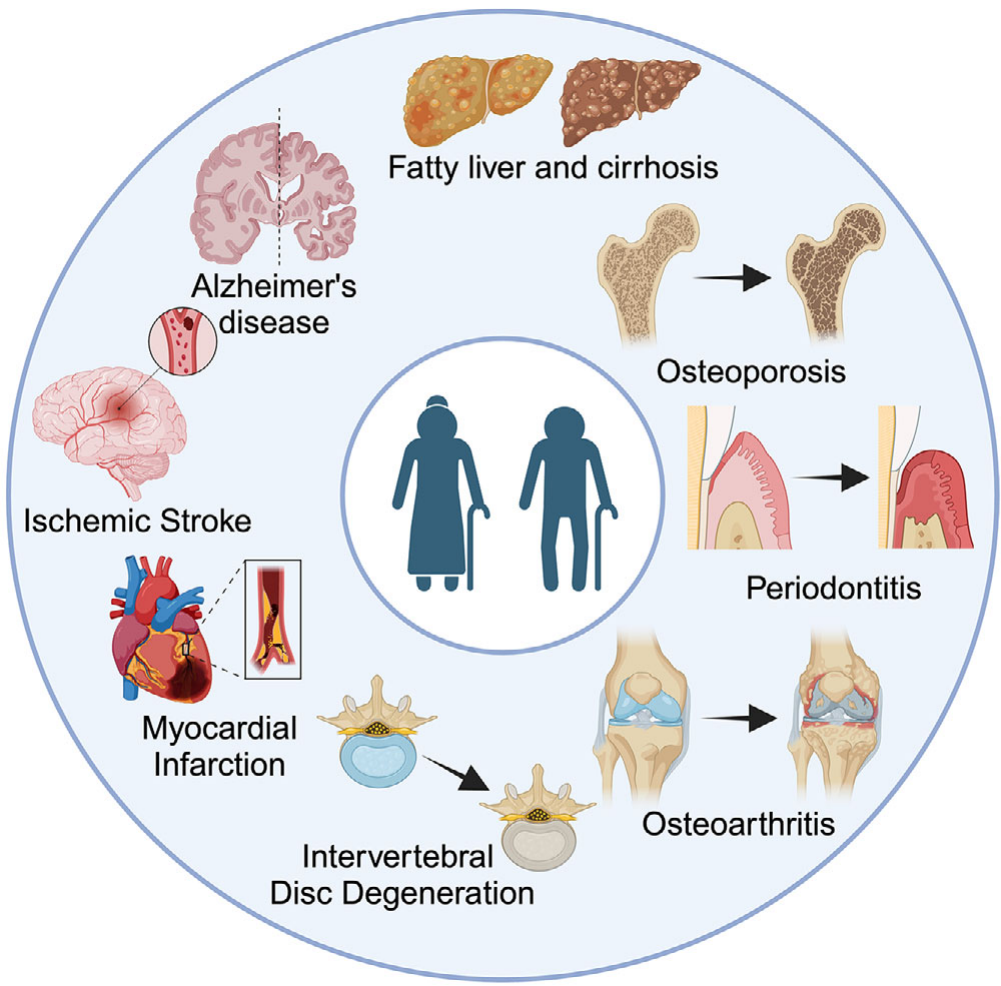
**Age‐related degenerative diseases**. Aging, characterised by a progressive decline of tissue, organ, and cellular functions, serves as a significant risk factor for a spectrum of multisystem degenerative disorders, encompassing cardiovascular diseases, neurodegenerative conditions like Alzheimer's disease, and musculoskeletal problems such as intervertebral disc degeneration, osteoporosis and osteoarthritis.

Improvements in the social economy of many countries together with advances in medical technology have greatly improved both the quality of life and the life expectancy of their populations. According to a report by the United Nations, the global population in 2022 was 8 billion, with the population 65 years of age or older projected to rise from 10% in 2020 to 16% in 2050, by which time the worldwide population ≥ 65 years would be more than twice as large as the population consisting of children ≤ 5 years and equal to that of children < 12 years.^1^ Population aging has thus become a major global problem. Aging is accompanied by a decline in the functions of tissues, organs, and cells.^2^ Moreover, aging is a risk factor for multi‐system degenerative diseases, including cardiovascular, neurodegenerative, and musculoskeletal diseases^3^ (Figure 1). Bone is a metabolically active tissue, and its regulation closely depends on a dynamic balance between resorption and formation. However, aging shifts this balance to favour resorption, leading to a loss of bone mass as well as the mechanical properties of bone, thus placing older adults at risk of injury.^4^

**FIGURE 2:**
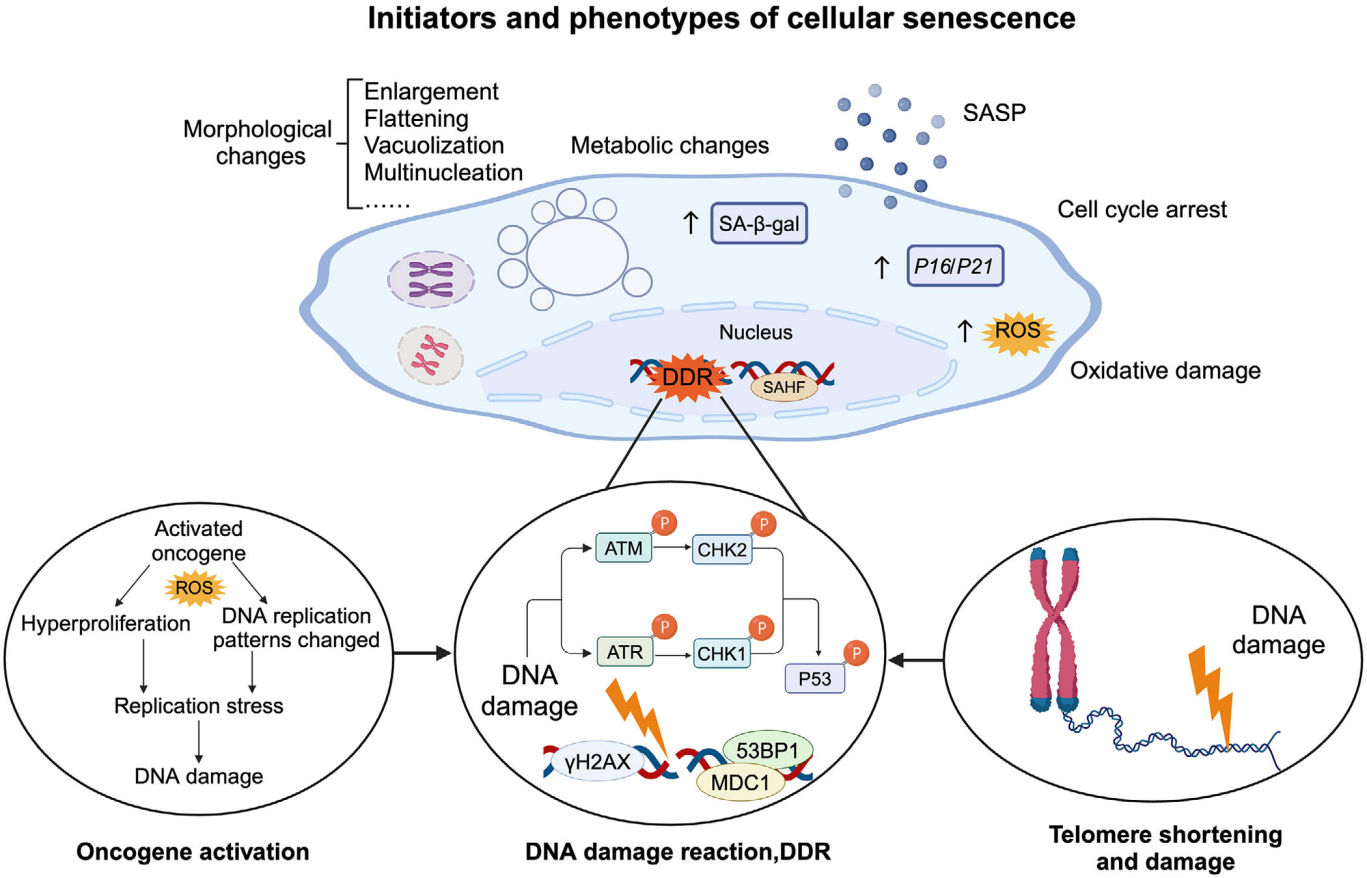
**Initiators and phenotypes of cellular senescence**. Various endogenous and exogenous stressors participate in the cellular senescence process. Telomere shortened in repeated cell divisions, oncogene activation, oxidative stresses, and radiation all lead to and accumulate nuclear DNA damages that could stimulate a signalling cascade of DNA damage response (DDR). The errantly prolonged DDR activation further triggers senescence. Once entered senescence establishment, multiple distinctions are triggered depending on the cell type and stimuli. Senescent cells undergo proliferative capacity loss, metabolic dysfunction, and common morphological changes including enlargement, flattening, vacuolisation, multinucleation, and disruption of nuclear membrane integrity. A variety of pro‐inflammatory factors, chemokines, and extracellular matrix remodelling proteases are secreted by the senescent cells to form a senescence‐associated secretory phenotype (SASP), which could hoard in the internal environment and may contribute to disorders via paracrine pathways. SASP, senescence‐associated secretory phenotype; SA‐β‐gal, senescence‐associated‐β‐galactosidase; DDR, DNA damage response; ROS, reactive oxygen species; SAHF, senescence‐associated heterochromatin foci; ATM, ataxia telangiectasia mutated.

Cellular senescence (CS), first proposed by Hayflick and Moorhead in 1961, refers to the progressive, irreversible loss of cell proliferative capacity caused by endogenous and exogenous stressors.^5^, ^6^ As a complex and heterogeneous biological process, CS exhibits significant heterogeneity in its triggers, phenotypes, and functional outcomes. Various triggers could initiate different types of CS. Telomere shortens in repeated cell divisions and leads to replicative senescence.^5^ Stressors like oxidative stress, DNA damage, and radiation cause stress‐induced senescence.^7^ Activation of oncogenes or loss of tumour suppressors triggers the oncogene‐induced senescence.^8^ Meanwhile, CS is considered a programmed process during embryonic development.^9^ Induced by diverse stimuli, distinct signalling pathways are activated during the process of CS.^7^, ^10^ Senescent cells undergo growth cycle arrest and dysfunction in parallel with morphological changes such as cellular enlargement, flattening, vacuolisation, multinucleation, and disruption of nuclear membrane integrity. Additionally, senescent cells secrete a variety of pro‐inflammatory factors, chemokines, and extracellular matrix remodelling proteases to form a senescence‐associated secretory phenotype (SASP)^11^ (Figure 2), whose composition and strength vary according to senescence duration, origin stimulus, and cell type.^7^ Under physiological conditions, SASP factors can activate the immune response and promote cell renewal and tissue regeneration. However, the homeostatic imbalance between the removal of senescent cells and the regeneration of new cells that occurs during aging leads to the accumulation of SASP factors in the internal environment as well as the promotion of CS via paracrine pathways and, over time, the development of senescence‐associated diseases.^12^

**FIGURE 3:**
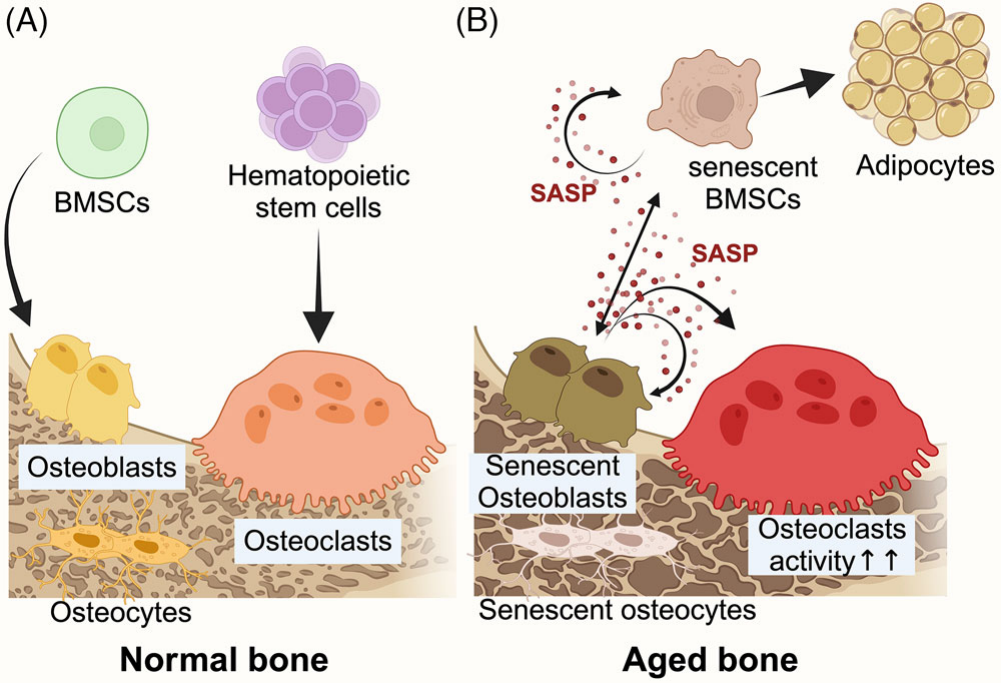
**Cellular changes in the skeletal aging microenvironment. (**A) In normal bone, BMSCs can differentiate into osteoblasts that can produce bone tissue. The newly generated bone could match the resorption of osteoclasts differentiated from the haematopoietic stem cells, keeping bone homeostasis. As bone tissue forms layer by layer, the osteoblasts are buried inside and transform into matured osteocytes. (B) During aging, BMSCs undergo senescence, and their proliferation and differentiation capacity are impeded, tilting BMSCs’ lineage into adipocytes. CS also hazards the osteogenic capacity of osteocytes. SASP secreted into the bone microenvironment could affect bone cells through paracrine and autocrine pathways and aggravate bone aging. BMSCs, bone marrow mesenchymal stem cells; SASP, senescence‐associated secretory phenotype; OBs, osteoblasts.

As individuals age, cells are exposed to various stressors that can trigger CS, while the accumulation of senescent cells in the microenvironment contributes to the collapse in organism function. The intimate interconnection between physical aging and CS gives us the possibility of antisenescence interventions towards aging‐related diseases.^13^ With dynamic metabolism, bone preserves bone mass and mechanical properties precisely with the balance between bone formation and resorption. The homeostasis in the skeletal system is mainly maintained by osteoblasts (OBs), differentiated from bone marrow mesenchymal stem cells (BMSCs), and osteoclasts (OCs) from the haematopoietic system. During aging, CS leads to a decline in osteogenesis due to a reduction in the osteogenic differentiation of BMSCs and the osteogenic capability of OBs^14^ (Figure 3).

Former research has examined several strategies to treat bone aging by delaying or reversing CS. These strategies include genetic approaches targeting genes or noncoding RNAs in BMSCs^11^, ^15^, ^16^ as well as pharmacological approaches (dasatinib, quercetin, rusolitinib).^17^ These efforts have been accompanied by the development of biomedical materials for degenerative bone disease treatment.^14^, ^18^, ^19^

**FIGURE 4:**
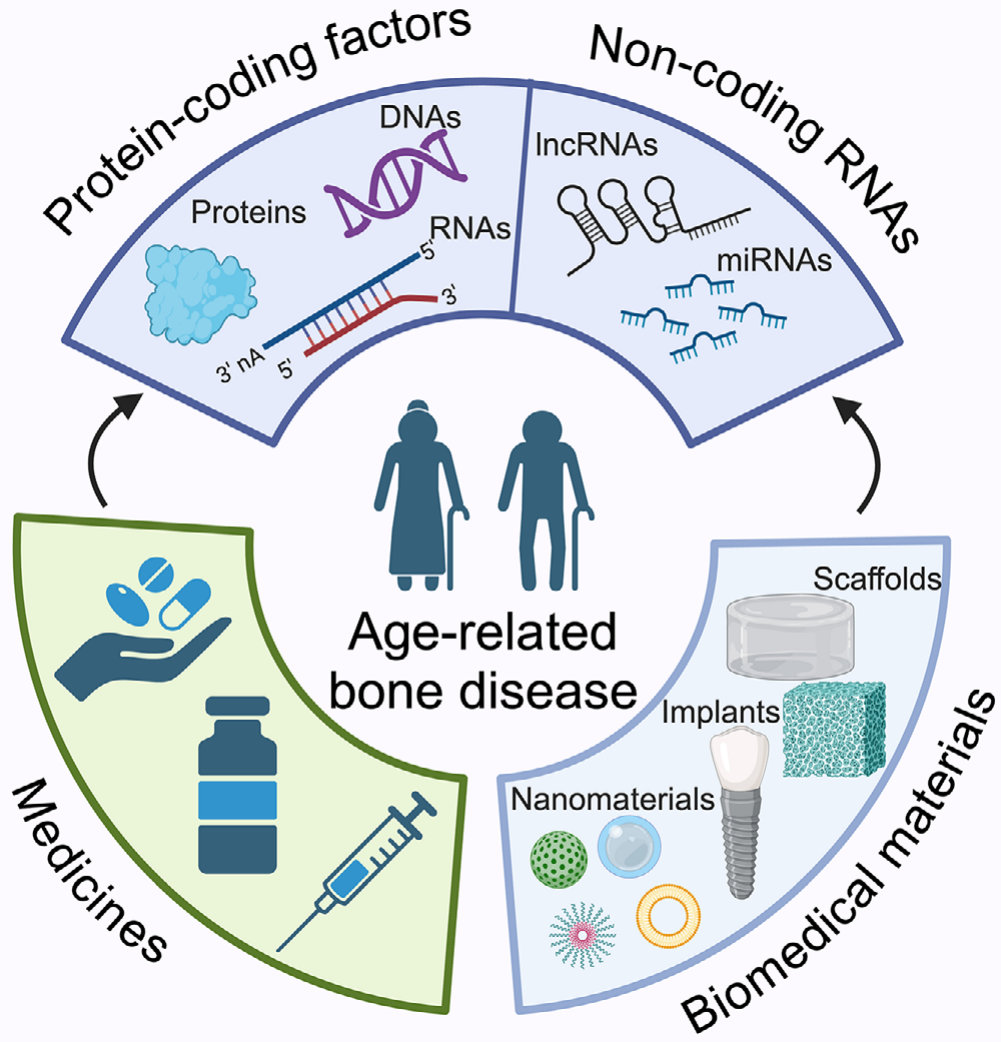
**Therapeutic strategies targeting cellular senescence in bone aging**. In this review, genes and noncoding RNAs (long noncoding RNAs, lncRNAs; micro RNAs, miRNAs) are reported as key anti‐CS targets against osteoporosis. Pharmacological agents and biomedical materials are also lay out as present and possible therapeutic strategies relieving CS through promoting osteogenesis. Together, anti‐CS treatment is considered as a promising approach against age‐related bone diseases.

In this review, we provide an up‐to‐date picture of bone aging research. We begin by examining the factors (genes and noncoding RNAs) responsible for mitigating cell aging and promoting osteogenesis and their potential as therapeutic targets. We then consider the pharmacological agents and biomedical materials being explored to prevent CS and relieve bone aging. The insights provided by our analysis contribute to supporting further research in the treatment and prevention of bone aging (Figure 4).

## 2 THERAPEUTIC TARGETS OF CELLULAR SENESCENCE IN BONE AGING

The development of treatments aimed at ameliorating bone aging includes targeting of the key factors involved in CS.

### 2.1 The role of protein‐coding factors in bone aging caused by cellular senescence

Proteins comprise the basic organic matter of cells and they mediate the processes essential for life. Accordingly, therapeutic strategies have focused on the proteins and thus also on the genes responsible for CS and bone aging^20^ (Table 1).

**TABLE 1:** Protein‐coding factors regulating cellular senescence and osteogenesis.

Factors	Functions	Interaction pathways	References
P16	Senescence marker genes	Control the G1 phase of the cell cycle Regulate CS, promote oxidative stress injury and osteoclast activity	Li et al.^21^
P53	Senescence marker gene	Regulate DNA damage and other stress responses ↑cell cycle arrest, ↑programmed cell death	Zheng et al.^22^
NAP1L2	↑CS ↓BMSCs’ osteogenic capacity	Activate the NF‐κB pathway Regulate SIRT to control H3K14 histone acetylation and inhibit osteogenic gene expression	Hu et al.^23^
Cav 1.3	↓proliferation, differentiation, autophagy, osteogenesis of BMSCs	↓Spred2	Fan et al.^27^
JAK	↑SASP secretion Participate in BMSCs’ CS	JAK2‐STAT3 pathway	Wu et al.^24^
USP10	↓P53 degradation, ↑transcription of CS factors	Regulate the deubiquitylation of P53	Wei et al.^25^
CHK2	↑p53, ↓BMSCs’ proliferation, differentiation, ↑CS, oxidative stress, DNA damage and SASP	Regulate pathways of DNA damage, mediate CS through p16/p19 regulation	Ji et al.^27^
SNF5	↓replicative senescence, ↑osteogenic differentiation of BMSCs	Bind to the *Oct4* promoter, maintain its H3K4me3 level and *Oct4* expression	Wu et al.^28^
YBX1	Regulate RNA selective splicing ↓BMSCs’ CS, ↑osteogenic differentiation of BMSCs	Correctly splice‐related transcript RNA	Xiao et al.^29^
SIRT1	↑bone formation in OBs, ↑osteogenic differentiation of BMSCs ↓oxidative stress in bone tissue, ↓BMSCs’ CS	Regulate the deubiquitylation of FOXO3a, ↑FOXO3a and SOD2 expression	Sun et al.^30^
IGFBP7	↓MSCs’ CS, ↑regenerative capacity of MSCs	Activate SIRT1, ↑H3K36ac deacetylation, ↓*P21* transcription	Li et al.^31^
JAG1	↑rapid expansion of MSCs ↑osteogenic potential of cells in vivo	JAG1‐Notch2‐Hes1 pathway ↑JAG1‐Notch pathway	Tian et al.^32^
BMP‐9	↓OBs’ CS, ↑bone mass in aged mice, ↑bone biomechanical properties	↓senescence gene expression, ↓SASP Smad1‐Stat1‐P21 axis	Xu et al.^33^
GDF11	↓MSCs’ CS, ↓age‐related bone loss	Smad2/3‐PI3K‐AKT‐mTOR pathway ↑DNA demethylase Tet2, ↑GDF11 promoter demethylation	Gao et al.^43^
LCN2 and PRL	↑BMSCs’ activity, ↓BMSCs’ CS, ↑preparations of osteogenesis and pre‐chondrogenic, ↑cranial bone defects repairing in mice		Tsai et al.^35^
BMI1	↑bone formation of OBs, ↓osteoclast activity, ↓ROS, ↓oxidative stress and DNA damage, ↓CS	1,25(OH)_2_D‐VDR pathway	Sun et al.^37^
NRF2	↑antioxidant signalling, ↓oxidative stress damage	VDR‐Nrf2‐Keap1 pathway	Yang et al.^36^
ANXA2	↑osteogenic differentiation, ↓MSCs’ CS in high glucose environments		Klabklai et al.^38^
SATB2	↓CS, ↑stemness, ↑osteogenic differentiation of BMSCs	Downstream regulatory target of estrogen	Wu et al.^39^
SCD2	↓CS, ↑stemness, ↑osteogenic and adipogenic differentiation of MSCs		Yu et al. ^40^
HDAC3	↓CS ↓SADS and TAFs, ↓adipogenesis in Runx2^+^ BMSCs		Yeo et al. ^41^
AMPKα1	↓MSCs’ CS, ↑osteogenic differentiation, ↓bone loss in a phospho‐mutant mouse model	AMPKα1/IGF‐1/CREB axis	Yang et al.^42^

↓, downregulate; CS, cellular senescence; ↑, upregulate; ROS, reactive oxygen species; NAP1L2, nucleosome assembly protein 1‐like 2; BMSCs, bone marrow mesenchymal stem cells; SASP, senescence‐associated secretory phenotype; USP10, ubiquitin‐specific protease 10; CHK2, checkpoint kinase 2; SNF5, sucrose nonfermentable 5; YBX1, Y‐box binding protein 1; SIRT1, Sirtuin 1; OBs, osteoblasts; FOXO3a, Forkhead box O3a; SOD2, superoxide dismutase 2; IGFBP7, insulin‐like growth factor binding proteins‐7; MSCs, mesenchymal stem cells; JAG, Jagged 1; BMP‐9, bone morphogenetic protein 9; GDF11, growth differentiation factor 11; LCN2, lipocalin‐2; PRL, prolactin; NRF2, nuclear factor erythroid 2‐related factor 2; BMI1, B lymphoma Moloney murine leukaemia virus (Mo‐MLV) insertion region 1; VDR, vitamin D receptor; ANXA2, annexin A2; SATB2, special AT‐rich sequence binding protein 2; SCD2, stearoyl‐CoA desaturase 2; HDAC3, histone deacetylase 3; SADs, senescence‐associated distention of satellite; TAFs, telomere‐associated foci; AMPKα1, adenosine monophosphate‐activated protein kinase alpha 1; CREB, cAMP‐response element binding protein.

#### 2.1.1 Genes that promote cellular senescence and inhibit osteogenesis

With increasing age, the expression levels of aging‐related factors, including those promoting CS, increase in bone cells, resulting in a decrease in bone formation.

The expression levels of CS‐associated genes (e.g., *P16*, *P53, NAP1L2*) in skeletal cells, especially MSCs, increase significantly with age, leading to rises in CS and falls in bone formation.^21^, ^22^, ^23^, ^24^, ^25^, ^26^, ^27^ These factors affect bone homeostasis through arresting the cell cycle, downregulating BMSCs’ and OBs’ function with multiple signalling pathways, including the JAK2‐STAT3 axis and the NF‐κB pathway.^23^, ^24^ Studies have proven that knockdown of *P16* and *P53*, both classic regulators of CS, ameliorates osteoporosis by inhibiting oxidative stress and other stress injuries, calming down excessive cell death, and reducing osteoclast activity.^21^, ^22^ NAP1L2, a node in the NF‐κB pathway, inhibits the expression of osteogenesis‐related genes through epigenetic regulation, while JAK affects CS and osteogenic differentiation by regulating the secretion of SASP factors.^23^, ^24^ In addition, proteins such as ubiquitin‐specific protease 10 (USP10) and Checkpoint kinase 2 (CHK2) play important roles in bone regeneration and antiaging by regulating P53 and its downstream targets.^25^, ^26^ Cav 1.3 negatively regulates Spred 2‐mediated autophagy and CS, thereby inhibiting the activity, osteogenic differentiation, and expression of key osteogenic markers in BMSCs, ultimately impairing their function.^27^ The detailed modes of action of each gene are shown in Table 1.

In summary, there are some proteins and their genes that promote bone aging and inhibit bone regeneration (Table 1). Therefore, targeted inhibition of the expression of these proteins and genes may restore bone homeostasis and promote bone formation. This provides a feasible direction for the future treatment of diseases related to bone aging.

#### 2.1.2 Genes that inhibit cellular senescence and promote osteogenesis

In terms of maintaining the stemness and osteogenic differentiation capacity of MSCs, several genes and their related pathways act positively to preserve bone homeostasis and regeneration (Table 1). Sucrose nonfermentable 5 (SNF5) binds to the promoter region of the *Oct4* gene and guarantees *Oct4* expression and osteogenic differentiation of BMSCs.^28^ Deletion of splicing factor Y‐box binding protein 1 (YBX1) leads to mRNA mis‐splicing, which triggers CS and impairs osteogenic capacity.^29^ Sirtuin 1 (SIRT1) and insulin‐like growth factor binding protein‐7 (IGFBP7) delay CS and promote osteogenesis by depressing oxidative stress and activating regenerative processes.^30^, ^31^ The Notch receptor ligand Jagged 1 (JAG1) prevents long‐term aging in vitro and potentially enhances BMSCs’ osteogenesis in vivo.^32^ Bone morphogenetic protein (BMP)‐9 and growth differentiation factor 11 (GDF11) modulate the SMAD protein family and its associated pathways to suppress the CS of OBs and MSCs and thereby relieve age‐related bone loss.^33^, ^34^ Lipocalin‐2 (LCN2) and prolactin (PRL) are similarly involved in regulating BMSCs’ activity and delaying CS.^35^ Pretreating BMSCs with LCN2 and PRL was shown to promote the repair of cranial defects in mice.^35^ Nuclear factor erythroid 2‐related factor 2 (NRF2) and polycomb protein B lymphoma Moloney murine leukaemia virus insertion region 1 (BMI1), downstream effectors of the vitamin D signalling pathway, promote the antioxidative signalling, clear ROS, and reset the balance of OBs and OCs activity, contributing to the antiosteoporosis function of vitamin D.^36^, ^37^ CS that occurs in specific environments, such as a high‐glucose environment or an estrogen‐deficient state, significantly affects the function and fate of BMSCs. Annexin A2 (ANXA2) restores the bone formation ability of BMSCs in the high‐glucose environment and alleviates the CS damage to their function.^38^ The estrogen‐sensitive special AT‐rich sequence binding protein 2 (SATB2) enhanced the stemness and osteogenic differentiation of BMSCs in the absence of estrogen.^39^ Stearoyl‐CoA desaturase 2 (SCD2) alleviates MSCs replicative CS and enhances osteogenic differentiation via the promotion of lipogenesis.^40^ The deletion of Histone deacetylase 3 (HDAC3) exacerbates CS and inhibits osteogenesis.^41^ Adenosine monophosphate‐activated protein kinase alpha 1 (AMPKα1) regulates bone loss through controlling CS and osteogenic lineage commitment of MSCs via the AMPKα1/IGF‐1 /CREB axis.^42^

Taken together, these genes and pathways play critical roles in bone aging and regeneration by inhibiting CS‐related genes, attenuating oxidative stress damage, and promoting cell proliferation and osteogenic differentiation (Table 1). These researches not only reveal the complex regulatory network between CS and bone homeostasis but also provide potential targets for developing therapeutic strategies against age‐related bone loss.

### 2.2 Role of noncoding RNAs in bone aging caused by cellular senescence

Noncoding RNAs (ncRNAs) are transcribed from the genome but not translated into proteins. They include long noncoding RNAs (lncRNAs) and microRNAs (miRNAs); both act as regulators of various cellular activities.^44^ With the development of innovative technologies such as microarrays and bioinformatics‐based assays, insights into the role and expression of noncoding RNAs in CS and osteogenesis have been obtained (Table 2).

**TABLE 2:** Noncoding RNAs regulating cellular senescence and osteogenesis.

Classification	Factors	Functions	Interaction pathways	References
lncRNA	*ZFAS1*	↑CS, ↓BMSCs’ osteogenic capacity	Sponge miR‐449 to upregulate EPHA5	Wu et al.^45^
	*MEG3*	↓BMSCs’ osteogenic differentiation, ↑postmenopausal osteoporosis	↑miR‐133a‐3p	Wang et al. ^46^
	*ENSRNOG00000056625*	↓BMSCs’ CS, ↑osteogenic capacity	Sponge miR‐1843a‐5p	Qi et al.^53^
	*LINC01638*	↓MSCs’ CS	Enrich on Chr22, ↑relative lncRNAs and proteins	Gordon et al.^15^
miRNA	miR‐34a	↓MSCs’ proliferation, ↓osteogenic differentiation, ↑CS	Target Nampt and mediate NAD^+^‐SIRT1 pathway	Pi et al.^11^
		↓ADSCs’ proliferation, ↓adipogenesis, ↓osteogenesis	↓cell cycle regulators, ↓transcription factors, ↓STAT‐3 expression and phosphorylation, modulate IL‐6 and ‐8 production	Park et al.^47^
		↓ADSCs’ proliferation, ↓cell viability, ↑CS	↓SIRT1	Mokhberian et al.^48^
	miR‐1292	↑ADSCs’ cS, ↓osteogenic differentiation in vitro, ↓bone formation in vivo	Wnt/β‐catenin pathway	Fan et al.^49^
	miR‐155‐5p	↓BMSCs’ proliferation and osteogenic differentiation, ↑apoptosis and CS	Hippo signalling pathway	Zhang et al.^50^
	miR‐29	↑skeletal CS, ↑SASP, ↓cortical bone thickness and bone mass	↑p53 via targeting Kindlin‐2 mRNA	Ding et al.^51^
	miR‐203‐3p	↓BMSCs’ cell growth, ↑CS, ↓osteogenesis	Bind to Pbk mRNA, ↓its expression, inhibit the ubiquitination‐mediated degradation of p53	Mei et al.^52^
	miR‐200c‐3p	↑MSCs’ CS, ↓stemness	bind to the 3'‐UTR of SCD2, ↓SCD2	Yu et al.^40^
	miR‐21‐5p	↓BMSCs’ CS, ↑osteogenesis of BMSCs, ↑angiogenesis of EPCs	AKT and ERK signalling pathway, pro‐angiogenic growth factors from EPCs	Qi et al.^54^
	miR‐196a‐5p	↓BMSCs’ CS, ↑osteogenesis and angiogenesis	Hoxa7/MAPK axis	Qi et al.^55^
	miR‐19a‐3p	↓OBs’	↓p16^Ink4a^, ↓p21^Cip1^	Kaur et al.^56^
	miR‐494‐3p	↑osteogenic differentiation of senescent BMSCs	PTEN/PI3K/Akt pathway	Yao et al.^57^

↓, downregulate; ↑, upregulate; lncRNAs, long noncoding RNAs; *ZFAS1*, zinc finger antisense 1; BMSCs, bone marrow mesenchymal stem cells; MSCs, marrow mesenchymal stem cells; miRNAs, microRNAs; ADSCs, adipose‐derived mesenchymal stem cells; SIRT1, sirtuin 1; STAT3, signal transducer and activator of transcription 3; SASP, senescence‐associated secretory phenotype; Pbk, PDZ‐linked kinase; SCD2, stearoyl‐CoA desaturase 2; EPCs, endothelial progenitor cells; MAPK, mitogen‐activated protein kinase; OBs, osteoblasts; PTEN, phosphatase and tensin homolog; PI3K, phosphoinositide 3‐kinase.

#### 2.2.1 Noncoding RNAs that promote cellular senescence and inhibit osteogenesis

Several ncRNAs have been shown to promote CS and to downregulate bone formation, by decreasing the viability of BMSCs.

The lncRNA *ZFAS1* (zinc finger antisense 1) binds to miR‐499 and upregulates the expression of ephrin type‐A receptor 5 (*EPHA5*), thereby tilting BMSCs’ transition towards adipogenesis. *EPHA5* knockdown inhibits CS, promotes autophagy in senescent cells, and increases bone mass in OVX mice.^45^ The lncRNA *MEG3* increases miR‐133a‐3p expression, thereby inhibiting the osteogenic differentiation of BMSCs, causing postmenopausal osteoporosis.^46^ The overexpression of miR‐34a in MSCs inhibits cell proliferation, blocks the cell cycle, promotes CS, and reduces the efficiency of osteogenic and adipogenic differentiation. These effects are reversed while using anti‐miR‐34a.^11^, ^47^, ^48^ miR‐1292 promotes CS, inhibits the osteogenic differentiation of adipose‐derived stem cells (ADSCs) in vitro, and delays bone formation in vivo via the Wnt‐β‐catenin pathway. By contrast, its suppression relieves CS and enhances osteogenesis.^49^ The increased levels of miR‐155‐5p that occur with aging inhibit BMSCs’ stemness and osteogenic differentiation while promoting apoptosis and CS by targeting Bmal1 and activating the Hippo signalling pathway.^50^ Ding et al. reported increased miR‐29 expression not only in the bone tissues of aged mice and osteoporosis patients but also in senescent MSCs. In addition, miR‐29 overexpression impairs bone health while elevating p53 and SASP levels. miR‐29 knockdown in BMSCs maintains bone mass and allows cranial defect regeneration.^51^ A recent study showed that overexpression of miR‐203‐3p in the BMSCs of young mice reduces cell growth and increases CS by preventing PDZ‐linked kinase (PBK) expression, hindering the degradation of p53.^52^

#### 2.2.2 Noncoding RNAs that inhibit cellular senescence and promote osteogenesis

Recent studies have identified noncoding RNAs that inhibit CS and promote bone tissue formation. Qi et al. found that lncRNA *ENSRNOG00000056625* alleviates aging and elevates the osteogenic capacity of BMSCs by binding to miR‐1843a‐5p, with the latter promoting senescence and negatively regulating osteogenesis.^53^ The same group demonstrated that a tetrahedral DNA (TDN)‐miR‐21‐5p nanocomplex mediates osteogenesis in aged BMSCs both directly, by activating the AKT and ERK signalling pathways, and indirectly by the secretion of pro‐angiogenic growth factors.^54^ In their study of cranial defects in aged rats, the authors showed that akermanite (Akt) bioceramics, containing Mg ions, mediate exosomal miR‐196a‐5p levels by targeting Hoxa7 and the MAPK pathway to prevent BMSC's CS and enhance bone regeneration.^55^ *LINC01638* initiates the osteogenic differentiation of MSCs and promotes their viability by directly targeting or modulating gene expression. A loss of *LINC01638* leads to MSCs’ senescence.^15^ Decreases in miR‐19a‐3p levels in both aged mice and humans have been observed. In H_2_O_2_‐induced senescent OBs, miR‐19a‐3p transcription correlates negatively with the degree of CS. Conversely, miR‐19a‐3p overexpression inhibits CS by downregulating p16^Ink4a^ and p21^Cip1^.^56^ A sharp decline in miR‐494‐3p was determined in senescent MLO‐Y4 cell‐derived exosomes whereas transfection with miR‐494‐3p restored expression in the exosomes and synergistically increased osteogenic differentiation in aged MC3T3‐E1 cells.^57^ As a result, different types of noncoding RNAs support bone homeostasis in the aging environment by inhibiting MSCs’ CS, enhancing oxidative stress resistance in OBs, and upregulating osteogenesis‐related genes.

NcRNAs play pivotal roles in regulating CS and osteogenic differentiation. Through aiming at key factors such as p53, p16, and p21 and regulating crucial pathways like Wnt/β‐catenin, Hippo, and PTEN/PI3K/AKT pathways, ncRNAs regulate CS, influence the fate of BMSCs, and alter the osteo‐adipogenesis balance. Their rise and fall consequently contribute to age‐related bone loss and osteoporosis, providing us with therapeutic promises.

**FIGURE 5:**
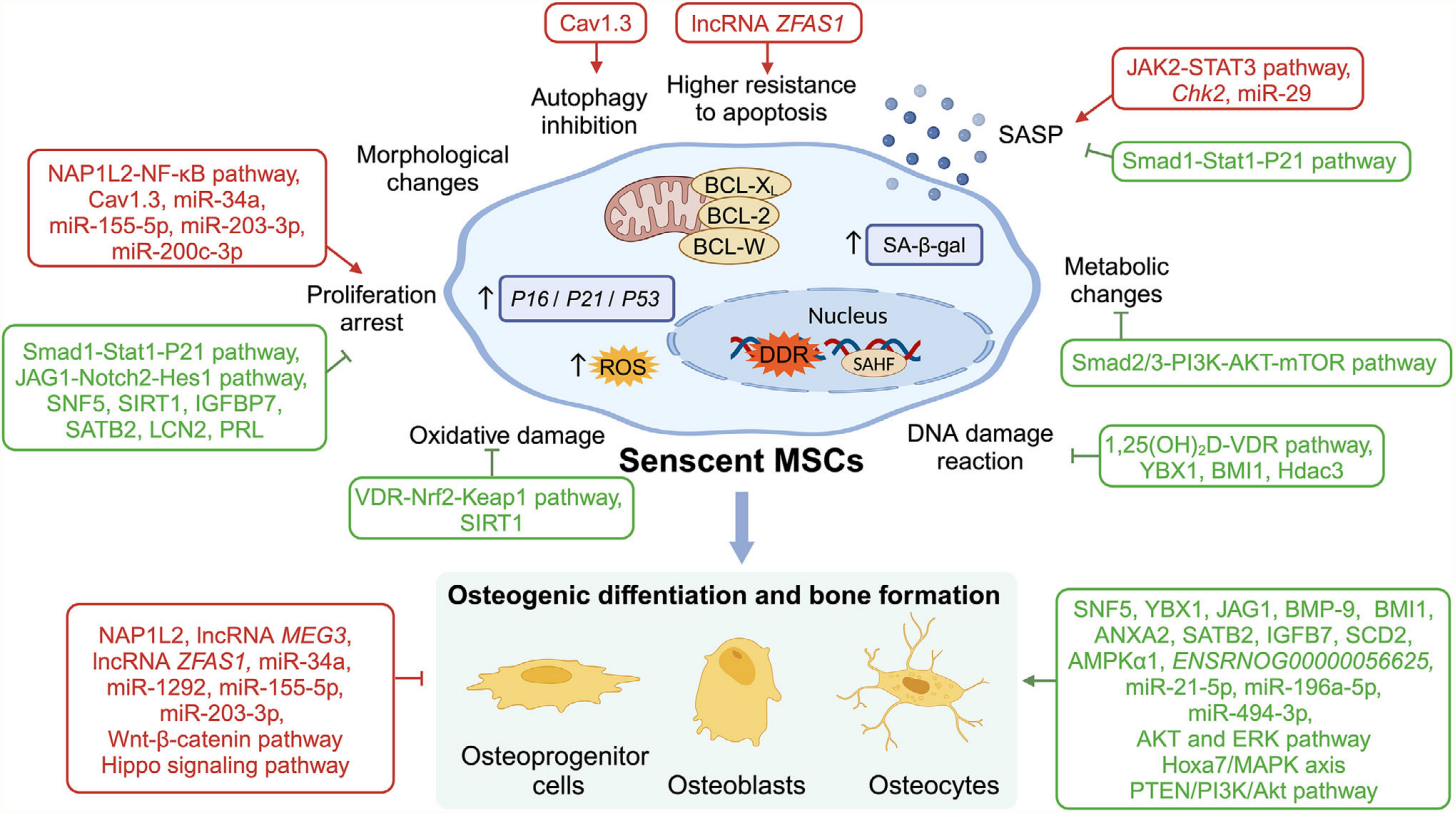
**Therapeutic targets of cellular senescence in bone aging**. Various genes and pathways play critical roles in bone aging and regeneration by inhibiting CS‐related genes, attenuating oxidative stress damage, and promoting MSCs’ proliferation and osteogenic differentiation. Through aiming at these possible targets, researchers may further discover novel treatments against age‐related bone disease through CS inhibition and osteogenesis promotion. DDR, DNA damage response; MSCs, mesenchymal stem cells; ROS, reactive oxygen species; SAHF, senescence‐associated heterochromatin foci; SASP, senescence‐associated secretory phenotype; SA‐β‐gal, senescence‐associated‐β‐galactosidase.

In summary, studies of CS and its relationship to stem cell proliferation and differentiation have begun to elucidate the mechanisms by which CS triggers age‐related bone loss (Figure 5). Both coding and noncoding genes play a role in this process. The results have provided an experimental and theoretical basis for the development of senescence‐targeting treatments aimed at preventing osteoporosis, bone fracture, and other age‐related bone diseases.

## 3 BONE‐PROTECTIVE MEDICINES AGAINST CELLULAR SENESCENCE

Since genetic engineering is thus far inaccessible for immediate clinical application, research efforts have been concentrated on finding novel agents and other therapeutic strategies to eliminate senescent cells or to mitigate the negative effects of CS on bone.

### 3.1 The inhibition of bone aging using senotherapeutics

Antiaging therapies targeting CS, or senotherapeutics, can be classified into two categories based on their mode of action: senolytics, which selectively purge senescent cells, and senomorphics, which prevent the detrimental cell‐extrinsic effects of senescent cells, such as SASP factors^6^ (Table 3).

**TABLE 3:** Established senotherapeutics with bone‐aging inhibitory effects.

Medicines	Functions	Interaction pathways	References
D+Q	Age‐preferential killing of senescent BMSCs, ↓expression of senescence‐associated and inflammatory markers, ↑osteogenic differentiation of senescent BMSCs, ↑cranial defects repairing in senescent mice	↓senescence‐associated antiapoptotic pathway	Zhou et al.^58^
	Systematically targeting senescent cells, reestablished cell cycle progression, ↑osteogenesis ability, ↓SASP	↑RAD51, ↑DNA repair capacity	Wang et al.^59^
	Ameliorate peri‐implantitis	↓pro‐inflammatory cytokines secreted by senescent cells	Yang et al.^60^
	Reverse glucocorticoid damage to the stemness and osteogenic potential of BMSCs	DPP4‐GLP‐1 axis	Wang et al.^61^
	Clear senescent cells, maintain mandibular condylar cartilage thickness, ↑subchondral bone volume and turnover	↓p21, Rela and senescence‐related Mmp13	Zhou et al.^62^
Fisetin	↑MSCs’ osteogenic differentiation, ↑OBs osteogenesis	GSK‐3β/β‐Catenin signalling pathway	Molagoda et al.^63^
	Eliminate aged BMSCs		Hambright et al.^64^
Navitoclax	Remove senescent cells, ↓cranial osteolysis in mouse model, ↓senescent macrophage SASP, ↓osteoclast formation	↓Bcl‐2 family protein activity in senescent cells	Su et al.^66^
Metformin	↓SASP secretion	NF‐κB signalling pathway	Di Micco et al.^6^
	In ADSCs: ↑osteogenesis‐related genes, ↑osteogenic differentiation, ↑intracellular SOD activity, ↓oxidative stress injury		Marycz et al.^68^
Rapamycin	In OBs: ↓SASP, ↓senescence markers, ↑OPG levels		Wang et al.^20^

↓, downregulate; ↑, upregulate; D+Q, Dasatinib**+**Quercetin; BMSCs, bone marrow mesenchymal stem cells; Rela, v‐rel avian reticuloendotheliosis viral oncogene homolog A; Mmp13, matrix metalloproteinase 13; OBs, osteoblasts; SASP, senescence‐associated secretory phenotype; ADSCs, adipose‐derived mesenchymal stem cells; SOD, superoxide dismutase; OPG, osteoclastogenesis inhibitory factor

The rejuvenating effects of several established antisenescence agents in bone aging have been reported. A cocktail made up of the senolytics dasatinib and quercetin (D+Q), which inhibit senescent cells by temporarily inhibiting senescence‐associated antiapoptotic pathways, was shown to age‐preferentially kill senescent BMSCs, reduce the expression of senescence and inflammatory markers, and stimulate senescent BMSCs to produce bone with more bone marrow space, similar to young BMSCs.^58^, ^59^ D+Q also ameliorated peri‐implantitis and reversed glucocorticoid damage to the stemness and osteogenic potential of BMSCs.^60^, ^61^ Additionally, D+Q was also applied in aged mice to ease the temporomandibular joint (TMJ) degenerative disorders.^62^ The senolytic fisetin was shown to promote stem cells’ osteogenic differentiation and OBs’ osteogenesis.^63^ In the clearance of senescent OBs, it was more effective than D+Q.^64^ Both D+Q and fisetin are being tested in an ongoing clinical trial investigating whether senolytics improve skeletal health in aged individuals.^65^ Navitoclax (ABT263) scavenges senescent cells, rejuvenates ultra‐high molecular weight polyethylene (UHMWPE) particle‐induced osteoporosis, and blocks OC formation by inhibiting SASP secretion from senescent macrophages.^66^ However, in addition to targeting senescent cells, Navitoclax significantly reduces the colony‐forming ability of BMSCs and impairs the calcified‐matrix‐forming ability of OBs.^67^ Therefore, the antisenescence and osteogenesis‐promoting effects of Navitoclax still need to be refined. Metformin, an NF‐κB signalling modulator, attenuates the production of SASP in senescent cells, thus acting as a ‘senomorphics’.^6^ Oral administration of metformin in healthy mice was shown to enhance the expression and secretion of BMP‐2, osteocalcin, and osteopontin in ADSCs, thereby improving their osteogenic differentiation capacity and significantly reducing oxidative stress to cells by boosting intracellular superoxide dismutase (SOD) activity.^68^ Wang et al. reported that rapamycin decreases the expression of osteoblastic SASP factors and senescence markers while upregulating osteoprotegerin (OPG) levels.^20^

### 3.2 Novel agents that inhibit cellular senescence and promote osteogenesis

Plants have been widely used as medicinal agents since ancient times and the efficacy of plant‐derived compounds in the treatment of tumours, atherosclerosis, depression, and other diseases has been investigated in clinical and experimental settings^69^, ^70^, ^71^ (Table 4).

**TABLE 4:** Novel medicines with senescence‐inhibitory and osteogenesis‐promoting effects.

Medicines	Functions	Interaction pathways	References
RSV	Reverse BMSCs’ CS	Mitofilin‐mediated promotion of mitochondrial function	Lv et al.^72^
	↓BMSCs’ CS, ↑osteogenic differentiation	AMPK‐ROS pathway	Zhou et al.^73^
	↑osteogenesis differentiation of BMSCs, ↓SASP, ↓oxidative stress damage	FAK‐AKT and MAPK pathways	Ali et al.^74^
Oleuropein	↑MSCs’ osteogenic differentiation ↓osteoblasts’ CS	↓connexin43 promoter activity, ↓gap junction intercellular communication	Varela‐Eirín et al.^75^
Glabridin	↑self‐renewal of MSCs, ↑osteogenic differentiation potential	↑OCT4, ↑DLX5, ↑RUNX2	Heo et al.^76^
Apigenin	↑BMSCs’ osteogenic differentiation, ↓CS, ↓oxidative stress	↑FAK and TGFβ	Ali et al.^77^
Proanthocyanidins	↑PDLSCs’ autophagy	PI3K/AKT/mTOR pathway	Liu et al.^78^
	↑PDLSCs’ osteogenic properties under inflammatory conditions	Wnt/β‐catenin pathway	Wu et al.^79^
Ginsenoside Rg1	↓BMSCs’ CS, ↑osteogenic and chondrogenic capacity of BMSCs	Antagonise the inhibitory effect of Licl on GSK‐3β to interfere with Wnt/β‐catenin pathway activation	Wang et al.^80^
Ginsenoside Rg3	↑BMSCs’ proliferation and differentiation potential, ↑osteogenesis	Enhance mitochondrial antioxidant capacity via Ca^2+^‐dependent pathway; ↑osteogenic gene transcription	Hong et al.^81^
Du‐Huo‐Ji‐Sheng‐Tang and Chuanxiong	↑MSCs’ osteogenic differentiation, ↓CS	Activate SMAD1/5/8 and ERK pathways to upregulate BMP‐2 and RUNX2 expression	Wang et al.^82^
TMP	↓LepR^+^ MSPCs’ CS	Ezh2‐H3K27me3 pathway	Gao et al.^83^
	Maintain the HSCs’ ecological niche, create an anti‐inflammatory, pro‐angiogenic environment in the bone marrow		
Rutaecarpine	↑BMSCs’ osteogenic differentiation, ↓CS, ↓oxidative stress	↑FAK and TGF‐β	Ali et al.^77^
Melatonin	↓BMSCs’ oxidative stress‐induced senescence, ↑osteogenic capacity	Activate AMPK‐SIRT1 pathway by melatonin receptor	Chen et al.^84^
	↑osteogenic differentiation of senescent BMSCs	Rebalance H3K36me2 and H3K27me3 modifications through MT1/2‐mediated NSD2 expression, ↑osteogenic gene	Xie et al.^85^
PTH	↓senescent cell accumulation, ↑MSCs’ proliferation, ↓SASP, improve bone microenvironment	↓Smad3 phosphorylation, ↓p16^ink4a^ promoter activity	Cui et al.^86^
ASTX	↓damage of bone marrow microenvironment, preserve HSCs and MSCs function, ↓CS, ↑promote haematopoietic development and bone formation in vivo	NRF2‐associated antioxidant defence system	Bhattarai et al.^87^
PQQ	Regulate redox balance, ↓osteoclast activity, ↓CS	↑antioxidant enzymes, ↑BMI1, ↓senescence‐related protein	Tang et al.^88^
SAM	↓CS, ↓ ROS, ↑osteogenic differentiation of MSCs	PI3K/AKT/FOXO3a axis	Shang et al.^89^
ED‐17	↓senescence‐related factors, ↓BMSCs’ CS and ROS levels	SIRT1‐Nrf2 pathway	Kou et al.^90^

↓, downregulate; ↑, upregulate; RSV, resveratrol; BMSCs, bone marrow mesenchymal stem cells; CS, cellular senescence; MSCs, mesenchymal stem cells; TMP, tetramethylpyrazine; ROS, reactive oxygen species; Vc, vitamin C; PDLSCs, periodontal ligament stem cells; FAK, focal adhesion kinase; SIRT1, sirtuin 1; PTH, parathyroid hormone; SASP, senescence‐associated secretory phenotype; ASTX, astaxanthin; CSDB, chondroitin sulphate‐derived biomaterial; ADSCs, adipose‐derived stem cells; PQQ, pyrroloquinoline quinone; SAM, S‐adenosyl‐l‐methionine; ROS, reactive oxygen species; ED‐17, eldecalcitol

Natural polyphenols, noted for their antiaging properties, have been applied in antiaging strategy exploration. In addition to Quercetin and other senotherapeutics mentioned above, novel polyphenolic substances have recently been proven to possess similar antiaging and osteoprotective properties. Resveratrol (RSV), found mostly in grape skins, is an activator of SIRT1 and exhibits antioxidant, anti‐inflammatory, and estrogenic activities. It has been shown to prevent osteoporosis by alleviating BMSCs’ senescence.^71^, ^72^, ^73^ It also promotes mitochondrial function in senescent stem cells, while inhibiting adipogenic differentiation, attenuating SASP and oxidative stress damage, and upregulating osteogenesis through the AMPK‐ROS, FAK‐AKT, and MAPK pathways to support bone homeostasis.^72^, ^73^, ^74^ Olive‐derived oleuropein enhances the osteogenic differentiation of MSCs in osteoarthritis; antiosteocyte senescence effects have also been described for other olive‐derived small polyphenols.^75^ Glabridin could enhance the self‐renewal capacity of MSCs and improve their osteogenic differentiation by upregulating the OCT4 gene.^76^ Apigenin, functionally screened from 143 natural compounds, could increase the osteogenic differentiation of BMSCs isolated from elderly female patients and raise the bone volume and cortical thickness of organotypic embryonic chick‐femur cultures ex vivo.^77^ Proanthocyanidins could renew PDLSC autophagy by downregulating the PI3K/AKT/mTOR pathway, and restore their osteogenic properties under inflammatory conditions through mediating the Wnt/β‐catenin pathway.^78^, ^79^ Besides, ginsenosides are a kind of chemical monomers derived from the Chinese herbal medicine Panax ginseng. Studies exploring the osteoprotective effects of senescence‐inhibiting plant‐derived natural products have shown that ginsenoside Rg1 can delay the senescence of BMSCs and improve their osteogenic and chondrogenic potential,^80^ while ginsenoside Rg3 enhances mitochondrial function and antioxidant capacity, increases the proliferation of BMSCs, prevents CS of BMSCs, promotes osteogenic differentiation, and retards lipogenic differentiation.^81^ Moreover, Wang et al. reported that Du‐Huo‐Ji‐Sheng‐Tang and its active component, Chuanxiong, stimulate osteogenic differentiation and retard the senescence of MSCs.^82^ Tetramethylpyrazine (TMP), the bioactive component extracted from Chuanxiong, improves the bone marrow microenvironment in aging mice and maintains bone homeostasis by inhibiting the senescence of LepR^+^ mesenchymal stem/progenitor cells, maintaining the haematopoietic stem cells niche, and inducing H‐type vessel formation.^83^ Rutaecarpine could upregulate OPG expression and played an osteoprotective role both in vivo and in vitro and has recently been investigated for its positive impact on osteogenic differentiation of BMSCs derived from older female patients.^77^ Plant‐derived natural products have shown great application prospects in anti‐CS and antiosteoporosis, but the specific mechanism and their clinical application still need further exploration. Future studies should focus on the molecular targets, signalling pathways, and long‐term safety assessment of these compounds in vivo.

Other natural substances derived from microorganisms, animals, and even humans have been explored for their ability to inhibit CS and, thus, their skeletal protective effects (Table 4). For example, melatonin was shown not only to attenuate BMSCs’ senescence and alter chromatin remodelling for genes involved in osteogenesis but also to protect bone mass in OVX rats.^84^, ^85^ Parathyroid hormone (PTH) attenuates senescent cell accumulation, enhances MSCs’ proliferation, reduces specific SASP levels in aging mice, and promotes bone reconstruction.^86^ The carotenoid astaxanthin protects the bone marrow microenvironment and stem cell function, inhibits CS, and promotes haematopoiesis as well as bone tissue formation in mice.^87^ Tang et al. showed that pyrroloquinoline quinone (PQQ) attenuates bone loss in periodontitis by regulating redox balance, decreasing OCs activity, and downregulating CS in osteoporotic mice.^88^ S‐adenosyl‐l‐methionine (SAM) is a critical intermediate in sulphur amino acid metabolism, and it was recently shown also to mitigate senescence, reduce ROS levels, enhance stemness in senescent MSCs, and improve bone structure in mouse models of premature aging. The mechanism of action underlying its antisenescence potential was shown to include PI3K/AKT signalling activation and the increased phosphorylation of forkhead box O3a (FOXO3a).^89^ Some natural product analogues with bone‐protective potential have been found to target CS, including the second‐generation active vitamin D analogue eldecalcitol (ED‐17), approved in Japan for the clinical treatment of osteoporosis. ED‐17 reduces the expression of senescence‐associated factors, ameliorates senescence in the BMSCs of OVX rats both in vivo and in vitro, and decreases ROS levels in BMSCs.^90^ These findings underscore the therapeutic potential of natural products and their analogues in combating age‐related bone disorders.

In summary, recent progress in the development of therapeutic agents targeting bone aging has mainly focuses on the targeted treatment of CS. They either reduce side effects by eliminating senescent cells or inhibiting SASP secretion from senescent cells, or inhibit bone resorption and promoting bone formation. These novel agents reduce the senescence load, restore the balance between bone resorption and bone formation, and alleviate age‐related skeletal decline. Therefore, they are not only expected to improve the prognosis of patients with age‐related bone diseases, but also point to the direction of subsequent research and development of drugs to treat bone aging.

## 4 BIOMEDICAL MATERIALS WITH ANTICELLULAR SENESCENCE PROPERTIES FOR BONE AGING THERAPY

The synergistic effect of biomaterials on existing medical treatment has been demonstrated in several settings. The use of biomaterials in antisenescence therapy and, thus, in the treatment of bone aging has accordingly aroused considerable research attention (Table 5).

**TABLE 5:** Biomedical materials for bone‐aging therapy targeting cellular senescence.

Category	Biomaterials	In vitro experiments	In vivo experiments	References
Scaffolding materials	Baghdadite ceramics	↓OBs’ CS, ↑proliferation and bone metabolic activity, ↑osteogenic gene expression in ADSCs	↑cranial defects repairing in aged rats	Lu et al.^18^
		Regulates crosstalk between ADSCs and OBs, ↑ADSCs’ osteogenic differentiation		Lu et al.^92^
	PDA‐JAKi/BGs@PCL	↓senescence markers in BMSCs, ↓SASP, ↑BMSCs osteogenic activity	↑cranial defects repairing in aged rats	Li et al.^14^
	High‐dosage H_2_‐releasing scaffold	Recruit BMSCs, ↓BMSCs’ CS, ↑regenerative capacity of BMSCs, ↑macrophage repolarisation	↑femoral defects repairing in aged rats	Chen et al.^93^
	OI‐EVs‐MBG functioned scaffolds	↓BMSCs’ CS, ↑osteogenic differentiation	↑bone regeneration in aged rats	Qi et al.^53^
	3D‐printed porous Mg‐containing Akt scaffolds	↑exosomal miR‐196a‐5p targeting Hoxa7 and activating MAPK signalling pathway	↑bone regeneration in cranial defects of aged rats	Qi et al.^55^
	TDN‐miR‐21‐5p@GelMA scaffold	↓BMSCs’ CS, ↑BMSCs’ osteogenesis, ↑EPCs’ angiogenesis via activating AKT and ERK signalling pathway	↑osteogenesis and angiogenesis in senescent critical‐size cranial defects	Qi et al.^54^
Implant surface modification	Sr‐SLA titanium implants	↑integrin β1, ↑spreading of senescent BMSCs, ↓ROS, ↓oxidative stress injury, lipogenic differentiation	↑osteointegration of tibial implants in aged rats	Zhou et al.^95^
	Ca‐PA titanium implants	↓BMSCs’ *P16*, *P21*, *P53* expression, ↓CS	↑femoral implant osseointegration, ↑peri‐implant bone quality in type II diabetic rats	Dong et al.^96^
	Bio‐MOF‐coated Ti implants (AHT‐Ce/SrMOF)	Activate AMPK signalling pathway in MSCs, ↓ROS, ↑DRP1, ↑MFN2, ↑OPA1, PINK1, ↑LC3, ↓CS	↓ROS, ↑osteogenic differentiation of MSCs, ↑osseointegration in OVX rat models	Chen et al.^97^
Nanomedicine delivery systems	Quercetin‐loaded senescence‐responsive TG‐18 hydrogel	Eliminate senescent BMSCs, ↑BMSCs’ self‐ renewal and osteogenic differentiation	↑senile mice femoral and cranial defects via eliminating local senescence, ↓MMPs secretion in bone	Tian et al.^101^
	Quercetin‐loaded (DSS)_6_‐liposomes	Eliminate senescent BMSCs	↓senescent cells in bone microenvironment, ↑BMSCs function, ↑senile mice bone formation	Tian et al.^102^
	DG‐NR‐Kre	↓MSCs’ CS, ↑osteogenic gene, ↑osteogenic differentiation	↑senile rats’ bone defects recovery, ↑new bone quality	Wang et al.^103^
	ZIF‐8/RSV	↓CS (OBs, BMSCs, VECs), obliterate intracellular ROS, ↑senescent OBs osteogenesis, ↑macrophage M2 polarisation		Xu et al.^104^
	HPB@RC‐ALN	↓ROS, ↓*RANKL* in senescent OBs, ↓OBs CS, ↓osteoclast differentiation	↓bone loss in OVX mice	Li et al.^105^
	PSeR hydrogel	↓ROS in senescent BMSCs, maintain DNA replication in an oxidative environment, ↑BMSCs’ antioxidant function, ↓CS, ↑self‐renewal	↑bone microenvironment, ↑bone defect regeneration, ↑healing of bone defects, ↑new bone quality in aging mice.	He et al.^106^
	TDN‐miR‐21‐5p@GelMA scaffold	↓BMSCs’ CS, ↑BMSCs’ osteogenesis and EPCs angiogenesis in aging environment	↑in‐situ vascularisation, ↑bone defect regeneration in aged cranial defect rat models	Qi et al.^54^
Other biomedical nanomaterials	CeO NPs	↓ROS, ↓DNA fragmentation, ↑*P53* expression, ↑autophagy and osteogenic differentiation of senescent BMSCs, ↓CS		Wei et al.^19^
	CeO nanozyme (CeO NP^60/40^)	↓ROS accumulation, ↓DNA damage, ↓BMSCs’ CS, ↓pro‐inflammatory and pro‐osteoclast markers, ↑osteogenic HIF1α release	↓bone structure decline, ↓bone loss induced by radiation damage in rats	Wei et al.^107^

↓, downregulate; ↑, upregulate; OBs, osteoblasts; CS, cellular senescence; ADSCs, adipose‐derived stem cells; BMSCs, bone marrow mesenchymal stem cells; SASP, senescence‐associated secretory phenotype; PDA, polydopamine; JAKi, Janus kinase inhibitors; BGs, bioglass nanoparticles; PCL, polycaprolactone; OI‐EVs, osteoinductive extracellular vesicles; MBG, mesoporous bioactive glass; SLA, sandblasted, large‐grit, and acid‐etched; ROS, reactive oxygen species; PA, phytic acid; Bio‐MOF, biofunctional metal−organic framework; ROS, reactive oxygen species; DRP1, GTPase dynamin‐related protein 1; MFN2, mitochondrial fusion protein; OPA1, optic atrophy 1; PINK1, PTEN‐induced putative kinase 1; LC3, light chain 3; TG‐18, triglycerol monostearate; MMP, matrix metalloproteinase; DG, D‐α‐tocopheryl succinate (DTS)‐Gal; NR, nicotinamide riboside; Kre, Kremen; ZIF‐8, zeolitic imidazolate framework‐8; RSV, resveratrol; VECs, vascular endothelial cells; HPB, hollow mesoporous silica nanoparticles Prussian blue nanozymes; RC, RANKL‐CRISPR/Cas9; ALN, alendrenate; OVX, ovariectomy; PSeR‐PEGS, PGA hydrogel loaded with SeR nanomicelles; EPCs, endothelial progenitor cells; CeO NPs, cerium oxide nanoparticles

### 4.1 Application of scaffold biomaterials to reverse cellular senescence in bone defect repair

Bone is a natural mineralised biomaterial that serves as a framework for the body. While the repair of large bone defects continues to challenge reconstructive surgeons, bioactive scaffolds have emerged as a potent tool in the treatment of bone regeneration.^91^

A novel bioactive baghdadite ceramic (Ca_3_ZrSi_2_O_9_) that prevents OB senescence and promotes OB proliferation and bone metabolic activity was developed by Lu's team. Compared with traditional hydroxyapatite/tricalcium phosphate (HA/TCP) scaffolds, baghdadite ceramic significantly induces osteogenic gene expression in ADSCs and demonstrates better efficacy in promoting cranial defect repair in rats.^18^ In co‐culture experiments, baghdadite ceramic was shown to modulate the crosstalk between ADSCs and OBs, increasing ADSCs’ osteogenic differentiation.^92^ Li and coworkers incorporated ruxolitinib, used to treat myelofibrosis, into a hierarchically biomimetic nanostructural electrospun three‐dimensional scaffold (PDA‐JAKi/BGs@PCL) that mimics natural extracellular matrix; it was also shown to inhibit the expression of senescence markers and SASP in vitro while enhancing cellular osteogenic activity. The two‐step release mechanism of PDA‐JAKi/BGs@PCL contributes to the sustained local delivery of ruxolitinib and prevents the bone resorption associated with post‐defect senescence.^14^ Chen and colleagues electrosprayed polyhydroxyalkanoate‐encapsulated CaSi_2_ nanoparticles onto mesoporous bioactive glass to obtain a high‐dose H_2_‐releasing scaffold. The local release of H_2_ was shown to recruit BMSCs, inhibit CS, and improve bone regeneration. It also led to the repolarisation of macrophages, thereby suppressing the pro‐inflammatory senescent microenvironment and promoting bone repair.^93^ Qi's group developed an osteogenic‐inductive extracellular vesicle mesoporous bioactive glass (OI‐EVs‐MBG) scaffold to improve bone regeneration by slowly releasing OI‐EVs. Their experiments demonstrated the ability of the OI‐EVs‐loaded MBG scaffold to stimulate osteogenesis, relieve BMSCs’ senescence in vitro, and enhance the repair of cranial defects in aged rats.^53^ A Mg‐containing Akt bioceramic was shown to promote both osteogenesis and angiogenesis in aged BMSCs by stimulating exosomal miR‐196a‐5p targeting Hoxa7 and activating the MAPK signalling pathway in vitro. Moreover, the 3D‐printed porous Mg‐Akt scaffolds strongly induced bone regeneration in the cranial defects of aged rats.^55^ Finally, excellent bone repair, with increased expression of osteogenic‐ and angiogenic‐related markers, in the in vivo repair of critical‐size cranial defects in aged rats was achieved using the above‐mentioned TDN‐miR‐21‐5p@GelMA scaffold developed by Qi's group.^54^

The remarkable potential of bioactive scaffolds in addressing age‐related bone diseases have been presented above. These innovative tissue engineering approaches demonstrated multifaceted therapeutic effects by simultaneously targeting CS, promoting osteogenesis, and modulating the impaired bone microenvironment, compensating for the lack of self‐healing ability of aged natural bone. Their biomimetic structures and functions also help replicate bone morphology, facilitate and stabilise bone regeneration in aging environment, providing solutions for bone fractures and defects with age‐related skeletal disorders.

### 4.2 Implant surface modification promotes osseointegration in senile patients

For bone implants, good clinical performance and long‐term prognosis depend on osseointegration of the implant surface into the bone. By shifting the fate of MSCs to favour the adipose lineage, CS substantially weakens bone formation and regeneration.^94^ Therefore, bone implantation therapy in aged individuals requires firm bonding between bone and implants. Zhou et al. applied sandblasted, large grit, acid‐etched (SLA) and hydrothermal methods to incorporate strontium (Sr) onto the surface of titanium implants. The Sr‐SLA coating was shown to upregulate integrin β1, promote the spread of senescent BMSCs, reduce CS by attenuating ROS levels and oxidative stress, and prevent the adipogenic differentiation of BMSCs, all of which improved osseointegration of the Sr‐SLA titanium implant.^95^ Phytic acid (PA) facilitates the connection between calcium ions and titanium, and calcium phytate (Ca‐PA)‐coated titanium implants were shown to downregulate *P16*, *P21*, and *P53* expression in BMSCs and to alleviate senescence stress in a high‐glucose environment. In addition, the rapid osseointegration of the Ca‐PA titanium implants in the femur of type II diabetic rats was observed, and the newly generated peri‐implant bone tissue was thicker and more homogeneous than in the SLA group.^96^ Chen's group developed a titanium implant with a biofunctional metal‐organic framework (bi‐MOF) coating by combining p‐xylylenebisphosphonate (PXBP) and Ce/Sr ions using a hydrothermal method. This innovative coating was able to remove ROS in MSCs, thereby restoring mitochondrial function and reversing CS. In vivo studies showed that the implants supported MSCs, promoted bone formation, and enhanced osteointegration in OVX rats.^97^

As discussed above, successful osseointegration of bone implants, particularly in aged individuals, is of great significance. CS impairs bone homeostasis and causes bone volume inadequacy around implants, leading to failure of implantation. Innovative surface modifications of implants have been developed to enhance osseointegration. These novel coatings reduce CS, attenuate oxidative stress, and promote osteogenic differentiation of BMSCs, shedding light on the potential of surface‐engineered implants to address age‐related challenges in bone regeneration and implant therapy.

### 4.3 Nanomaterials targeting cellular senescence used in bone disease therapy

Nanotechnology and its derivatives have brought innovation to tissue engineering and bone healing. Many of the new biomedical nanomaterials currently being used in orthopaedic trauma and repair are also being explored for their therapeutic efficacy in bone diseases^98^ and have yielded promising results.

#### 4.3.1 Nanotherapeutics delivery systems

The dense structure and single blood supply of bone hinder drug distribution in the tissues, such that higher doses and longer treatment duration are often needed,^99^ including for senotherapeutics. The development of bone‐tissue‐targeted therapeutic delivery systems would increase medicine bioavailability and biodistribution, thus improving efficacy while reducing side effects.^100^

Tian's group prepared quercetin‐loaded senescence‐responsive triglycerol monostearate (TG‐18) hydrogels to target the increased secretion of matrix metalloproteinases (MMPs) by senescent cells and thus eliminate senescent BMSCs in bone defects and promote bone regeneration in older adults.^101^ They also applied liposome‐encapsulated quercetin to enhance medicine solubility and modified it with bone‐targeting properties using a bone‐affinity peptide (DSS)_6_. When compared with conventional delivery, this system resulted in better senescent cell clearance and a greater therapeutic effect in senile osteoporosis.^102^ Liu et al. synthesised a D‐α‐tocopheryl succinate (DTS, hydrophobic)‐Gal (hydrophilic) (DTS‐Gal, DG) amphiphilic diblock copolymer, encapsulating RSV in its core and binding nicotinamide riboside (NR) on its shell, to achieve dual loading. By linking the anti‐Kremen1 antibody to the micellar shell and hydrolysis of the hydrophilic chain triggered by senescence‐associated β‐galactosidase (SA‐β‐Gal), the specific responsive release of NR and RSV from DG‐NR‐Kre was achieved; excellent biocompatibility, antiaging, and bone‐enhancing properties were demonstrated in vivo and in vitro.^103^ Wang et al. loaded RSV into a zeolitic imidazolate framework‐8 (ZIF‐8/RSV), which amplified the pro‐proliferation, ROS scavenging, and osteogenic differentiation effects of RSV in senescent cells. ZIF‐8/RSV was also shown to enhance osteogenesis in the senescent environment by modulating macrophage M2‐type polarisation and promoting vascular regeneration.^104^ By greatly improving the precision of antiaging medication, these novel drug delivery systems not only contribute to the efficacy and therapeutic effect of senotherapeutics but also increase the safety of the delivered agents and reduce the occurrence of side effects.

The excessive accumulation of ROS, one of the hallmarks of CS, leads to oxidative stress and to the deterioration of the tissue microenvironment, which together hinder the repair of bone defects in the elderly.^105^ Li and coworkers constructed a novel composite nanoparticle, HMSN‐PB, consisting of Prussian blue nanozyme (PBzyme) generated in situ on the surface of hollow mesoporous silica nanoparticles (HMSN). The mesoporous structure of HMSN‐PB allowed its combination with a RANKL CRISPR/Cas9 (RC)‐expressing plasmid and the bone‐targeting agent alendronate to form HMSN‐PB@RC‐ALN. The construct was shown to inhibit OB senescence, prevent OC activation, and alter the osteoporosis microenvironment by scavenging ROS and reducing RANKL gene expression in senescent OBs.^105^ Liu's group prepared injectable p PEGylated poly (glycerol sebacate) (PEGS‐NH2)/poly (γ‐glutamic acid) (γ‐PGA) hydrogel containing rapamycin‐loaded poly (diselenide‐carbonate) nanomicelles (PSeR) to form a multi‐dimensional ROS‐scavenging system. When used to treat senescent BMSCs, the hydrogel exhibited high‐sensitivity ROS responsiveness, as the dynamic release of the rapamycin‐loaded nanomicelles led to the scavenging of the accumulated intracellular ROS.^106^ Among the other demonstrated features of PSeR were the preservation of DNA replication in an oxidative environment, regulation of the antioxidant function of BMSCs, the delay of CS, self‐renewal of BMSCs, and in vitro and vivo osteogenesis.^106^ Hence, by targeting senescent osteal cells, the antioxidant activity of the medicine delivery system attenuated oxidative stress in the senescent microenvironment, inhibited CS, and maintained the function of osteal cells across multiple pathways.

In conclusion, nanodelivery systems can enhance the stability of therapeutic agents, improve their bioavailability, achieve synergistic effects through dual or multi‐agent loading, ensure appropriate biodistribution, reduce unwanted side effects, and thus improve the efficiency of treatment. The application of these systems to senotherapeutics will achieve intelligent drug release and the targeted elimination or inhibition of senescent cells in the skeletal microenvironment, allowing the restoration of bone homeostasis and, therefore, of bone tissues. The above results also indicate that the nanodelivery system has great potential in the field of bone aging treatment.

#### 4.3.2 Other biomedical nanomaterials

Besides the delivery systems, nanoparticles have other applications in bone senescence therapy by targeting CS. For example, cerium oxide (CeO) NPs possess antioxidant and pro‐regenerative properties and can be taken up by cells. Under normal culture conditions, CeO NPs reduce CS and significantly enhance autophagy, osteogenesis, and bone matrix deposition. Pretreatment of BMSCs with CeO NPs results in ROS scavenging, a reduction in DNA fragmentation, an increase in P53 expression, the regulation of autophagy activity, and attenuation of the damage of ionising radiation.^19^ Based on these findings, an artificial nanoenzyme composed of CeO was developed and its prevention of ionising‐radiation‐induced bone loss in rats was explored.^107^ Among the demonstrated effects of the nanozyme were ROS scavenging, the prevention of both DNA damage and CS, a reduction of OC activity, and the regulation of both macrophage‐derived OCs and bone progenitor cells.^107^ Through their unique antioxidant and pro‐regenerative properties, CeO NPs no doubt offer a novel therapeutic strategy for targeting CS and promoting bone formation.

In summary, innovative biomedical materials are being developed to promote bone tissue health and regeneration by interfering with the CS process. These innovative products are expected to provide a new approach to the treatment of bone aging. However, the relevant research is still in its infancy, and additional basic and clinical studies of the safety and efficacy of these materials are required. In addition, the standardisation of the synthesis of these biomaterials is also an issue that needs to be focused on in the future.

## 5 DISCUSSION

Given recent research advances, new medicines and delivery systems that target CS and thus prevent or reverse bone aging and other age‐related degenerative diseases can be expected in the near future. In 2017, Farr and colleagues demonstrated the role of CS in age‐related bone loss and experimentally validated the protective effects of senotherapeutics on the aged skeleton.^17^ In their 2020 review, the authors focused on the mechanism by which skeletal CS triggers age‐associated bone loss and on the potential of antisenescence therapy in bone aging.^108^

The regulatory network that links CS to bone aging is gradually being elucidated in studies of the changes in age‐related gene expression and, thus, in the respective RNA and protein levels. He et al. systematically investigated the molecular mechanisms of CS and laid out the main signalling pathways in this procedure.^109^ With further research documented, our present review added up several other relevant cellular signalling pathways, including Notch, PI3K‐AKT‐mTOR, and JAK2‐STAT3, and their activation by various effector proteins. Moreover, the roles of noncoding RNAs, such as lncRNAs and miRNAs, in the determination of cell fate are also elaborated. The impact of current epigenetics hotspots (DNA methylation, histone modification, etc.) on anti‐CS and osteogenesis is also considered. The studies discussed herein suggest that regulating the key factors involved in the cellular regulatory network can restore the age‐compromised osteal microenvironment by re‐establishing the balance between bone formation and bone resorption and promoting osteogenesis to facilitate the reconstruction of bone defects or reverse osteoporosis. Though the existing results are lack of sufficiency to constitute a perfect cellular senescence regulatory network, the insights obtained in these studies still establish a remarkable basis for new therapeutic options in the treatment of age‐associated bone loss.

**FIGURE 6:**
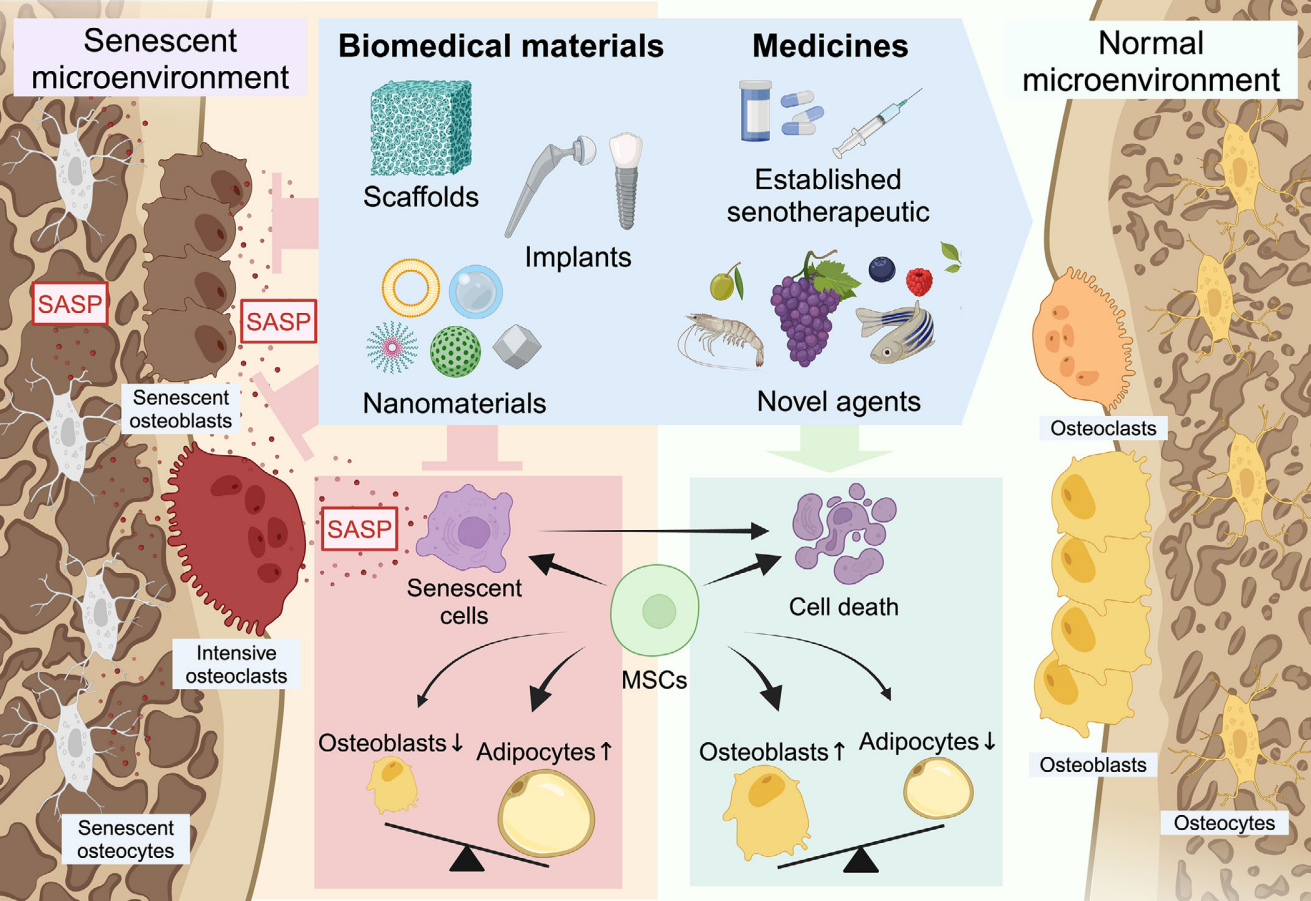
Therapeutic pathways for bone‐aging targeting cellular senescence. The senescent microenvironment not only impairs the regenerative and differentiation capacity of MSCs, tilting the balance of the cell lineage toward adipogenic differentiation but also impedes osteoblast bone formation capacity and increases osteoclast activity. With novel therapeutic agents and biomedical materials applied, CS and its concomitant phenotypes are alleviated, reversing the normal differentiation balance and transferring the microenvironment into a healthier state. MSCs, mesenchymal stem cells; SASP, senescence‐associated secretory phenotype.

Our review also considered the links between CS and degenerative bone diseases as well as the therapeutic potential of this relationship. Senotherapeutics such as D+Q, Navitoclax, and metformin have shown valid therapeutic effects in bone, especially the D+Q and Fisetin, which are being validated in clinical trials conducted against age‐related bone loss.^65^, ^110^ There is also growing interest in plant‐derived compounds. The desirable effects of several plant‐derived natural products, mainly ginsenosides and RSV, have been reported, suggesting their use as anti‐inflammatory agents, inhibitors of oxidative stress damage, and promoters of cell proliferation and osteogenic differentiation. Melatonin, PTH, vitamin D, and other readily available natural products have shown efficacy in reversing CS and improving bone mass; however, most of the promising results have been obtained in in vitro studies, such that the biosafety and efficacy of anti‐CS bone‐protecting medicines still need to be demonstrated in animal experiments and clinical trials (Figure 6).

Recent innovations in biomedical materials offer several approaches to CS‐targeted bone‐aging therapy. Scaffolds based on bioactive ceramics and polymeric biomaterials were shown to significantly improve bone defect repair in animal aging models, with the material itself or its medicine cargo inducing therapeutic effects. Similarly, the implant surface can be modified to exhibit anti‐CS properties, to improve the long‐term outcome of implant restoration in older patients. Nanomaterials have also been examined for their effects on bone senescence, including the use of hydrogels, liposomes, and nanomicelles as delivery vehicles for senotherapeutics. With the possibility of precise, tissue‐specific delivery, these systems will greatly improve the bioavailability, efficiency, and safety of the carried drugs. Moreover, the anti‐CS properties of CeO NPs and the construction of the corresponding nanozyme offer new strategies for bone restoration and the treatment of bone injuries (Figure 6).

However, much remains to be learned about the CS regulatory network and only a few medicines have been successfully combined with advanced biomedical materials, such that the potential of this approach remains largely untapped. Most research is still dominated by in vitro experiments, and animal experiments are often conducted on small animals. A better understanding of the regulatory targets of CS and the related signal pathways is needed to develop effective therapeutic agents and delivery systems for the treatment of bone aging. In this regard, combination therapy consisting of anti‐CS agents and biomedical materials currently seems to hold promise. Despite the significant progress in CS understanding and its role in bone aging, the lack of large animal models for CS‐related bone loss studies is one of the major limitations in the field. Large animals such as sheep, pigs, and nonhuman primates are vital models for translating basic research into clinical applications, owing to their similarity to humans in size, structure, and function.^111^, ^112^ However, the application of large animal models is limited due to not only the high expense in time and costs but also the strict ethical and regulatory constraints. To overcome such limitations, D‐galactose and radiation can be used to induce aging in animal models, meanwhile, building progeria models could help accelerate aging symptoms for in vivo experiments.^113^, ^114^, ^115^ On the other hand, with the continuous development of organoid technology, we believe that more and more bone organoids will be used to study bone aging in the future.^116^, ^117^ On this basis, clinical trials shall be further run to confirm the safety and efficacy of senolytics in bone aging treatments targeting CS.

Although this article summarises the latest progress in the field of bone aging treatment, there are still some limitations. Among the limitations of this review are its reliance on PubMed as the main literature search library and the main search terms set as: (senescence[Title/Abstract]) AND ((bone[Title/Abstract]) OR (osteo[Title/Abstract])) AND ((gene[Title/Abstract]) OR (medicine[Title/Abstract]) OR (biomaterials[Title/Abstract])). The inclusion only of publications from the English medical literature may also lead to the omission of relevant studies. In addition, most of the studies consisted of basic research, and their findings need to be further validated in clinical trials. Furthermore, their results were mostly qualitative, which hinders horizontal comparisons. This makes it impossible to quickly select the most effective treatment. Finally, in terms of therapeutic approaches, our review was largely limited to an examination of the effects of certain factors, drugs and biomaterials, with insufficient attention given to combined therapeutic modalities. However, these limitations also provide directions for future research. For example, conduct large animal experiments or clinical trials, and conduct combination therapy of factors, drugs and biological materials.

**FIGURE 7:**
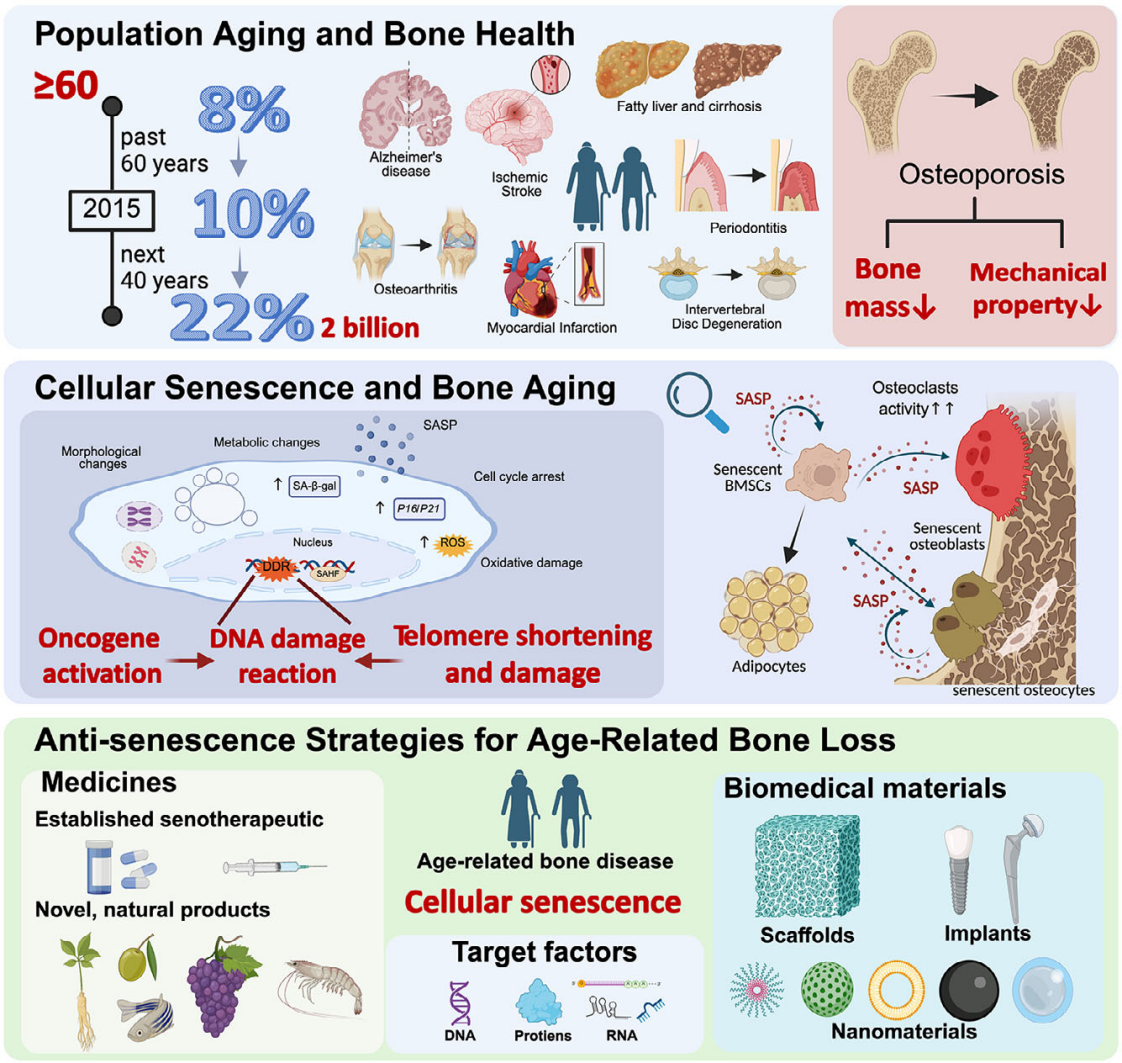
**Cellular senescence in bone aging and the antisenescence therapies for age‐related bone loss**. With extended lifespans causing population aging, bone aging has become a worldwide issue. Cellular senescence in the bone microenvironment causes bone degeneration and dysfunction. Continuing progress in studies of the relationship between senescence and bone aging will lead to effective strategies for the prevention, treatment, and prognosis of age‐related bone diseases.

Generally speaking, with extended lifespans leading to population aging, bone aging has emerged as a global concern. Cellular senescence within the bone microenvironment is a key driver of bone degeneration and dysfunction. There is no doubt the continuing progress in studies of the relationship between CS and bone aging will lead to effective strategies for the prevention, treatment, and prognosis of age‐related bone diseases such as osteoporosis, bone trauma in older individuals, and edentulism, as well as age‐related functional deterioration of other organs and systems (Figure 7).

## AUTHOR CONTRIBUTIONS

**Q. Zhu**: Conceptualisation, Writing – original draft, Visualisation. **M. Hu**: Conceptualisation, Writing – review & editing. **L. Wu**: Writing – review & editing. **E. Wei**: Writing – review & editing. **X. Pan**: Writing – review & editing. **H. Liu**: Supervision. **Y. Liu**: Writing – review & editing, Supervision. All authors contributed to the manuscript and approved the submitted version.

## FUNDING

This study was supported by the National Natural Science Foundation of China (82370924, 82170929), the Beijing Natural Science Foundation (L222090, L222145), and the Fujian Province Natural Science Foundation of China (2021J01803).

## CONFLICT OF INTEREST STATEMENT

The authors declare that they have no known competing financial interests or personal relationships that could have appeared to influence the work reported in this paper.

## ETHICS STATEMENT

This review synthesizes previous studies and does not involve any original data collection from human participants or animals. Therefore, ethical approval was noy required.
